# Effect of Chlorogenic Acid Intake on Cognitive Function in the Elderly: A Pilot Study

**DOI:** 10.1155/2018/8608497

**Published:** 2018-03-07

**Authors:** Morimasa Kato, Ryuji Ochiai, Kazuya Kozuma, Hirotaka Sato, Yoshihisa Katsuragi

**Affiliations:** ^1^Department of Health and Nutrition, Yamagata Prefectural Yonezawa University of Nutrition Sciences, Yonezawa 992-0025, Japan; ^2^Health Care Food Research Laboratories, Kao Corporation, Tokyo 131-8501, Japan; ^3^R&D, Development Research, Health Care/Household/Chemicals, Kao Corporation, Tochigi 321-3497, Japan

## Abstract

**Objective:**

To evaluate the effect of chlorogenic acids (CGAs) intake on cognitive function.

**Methods:**

In this pilot study, the Cogstate and CNS Vital Signs test batteries were used to evaluate cognitive function in 8 healthy elderly men and women complaining of subjective memory loss after a 6-month intake of a test beverage containing 330 mg of CGAs just before bedtime.

**Results:**

After a 6-month CGA intake period, significant improvement was observed in the One Back Test of the Cogstate, the Shifting Attention Test, and Finger Tapping Test as well as in the composite memory, verbal memory, complex attention, cognitive flexibility, executive function, and motor speed domains of the CNS Vital Signs test battery.

**Conclusion:**

A 6-month intake of CGAs may improve attentional, executive, and memory functions in the elderly with complaints of subjective memory loss.

## 1. Introduction

Aging is a biological process that occurs in all life forms and is accompanied by adverse effects on physical and cognitive functions in humans. The incidence of dementia, a disease strongly associated with cognitive impairment, is on the rise globally. Alzheimer's Disease International, a worldwide federation of Alzheimer's associations, estimates that the number of patients with dementia is expected to double every 20 years [1]. Early prevention of dementia is critical because no definitive therapy has been established. One study reported an association between lifestyle factors and the onset of dementia [2]. Other studies have proposed that certain foods, a key lifestyle factor, contain nutrients that prevent dementia [3, 4].

High intake of fruits, vegetables, fish, nuts, and legumes and low intake of meat, high fat dairy, and sweets have been shown to delay aging-related cognitive impairment and reduce the risk of Alzheimer's disease (AD) [5]. The mechanism underlying the amelioration of cognitive impairment and dementia via fruits and vegetable intake is likely mediated by antioxidants and antioxidant vitamins [4]. In addition, the intake of n-3 polyunsaturated fatty acids (PUFAs), which are abundant in fish, has been reported to reduce the incidence of cognitive impairment and dementia [3]. Docosahexaenoic acid was found to reduce amyloid *β* (A*β*) deposition and to exert neuroprotective and anti-inflammatory effects at synapses in a mouse model of AD [6]. Additionally, a cross-sectional study involving dementia-free participants in the Framingham Study demonstrated that middle-aged and elderly participants who had a low red blood cell concentration of n-3 PUFAs also had small brain volumes and low cognitive function [7]. Studies have also reported an association between consumption of large amounts of green tea or coffee and reduced risk of dementia [8, 9].

Improved cognitive function as a result of drinking coffee is thought to be mediated by caffeine and chlorogenic acids (CGAs). A study conducted in rats showed that caffeine mediates long-term potentiation in the Cornu Ammonis 1 area of the hippocampus with suppression of A*β* aggregation [10]. In addition to caffeine, coffee contains CGAs and has antioxidant properties [11]. The CGAs in coffee beans are composed of 9 major compounds: 3 caffeoylquinic acids (5-CQA, 3-CQA, and 4-CQA); 3 dicaffeoylquinic acids (3,4-diCQA, 3,5-diCQA, and 4,5-diCQA); and 3 feruloylquinic acids (3-FQA, 4-FQA, and 5-FQA). 5-CQA (formerly called 3-CQA or chlorogenic acid) is the main component of CGAs in roasted and green coffee beans.

Studies using mice and cultured neurons have shown that CGAs protect neurons and suppress the aggregation of A*β* through antioxidative effects [12, 13]. Furthermore, caffeic acid, coumaric acid, ferulic acid, and sinapic acid, all of which are metabolites of CGAs, have been shown to improve cognitive function [13–15]. These findings suggest that CGA intake contributes to improved cognitive function, but the mechanism by which CGA intake affects cognitive function in humans is currently unknown.

In this study, we hypothesized that CGAs improve human cognitive function, especially in the elderly, and sought to elucidate the association between CGAs and cognitive function from the perspective of preventing dementia, the incidence of which increases with aging in this population. Therefore, in this study, we enrolled community-dwelling elderly individuals to examine the effects of a 6-month intake of CGAs on cognitive function and the association with biological data.

## 2. Methods

### 2.1. Participants

For recruitment of the participants, an explanatory meeting for this study was held at a regional community center in Yonezawa city, Yamagata, Japan, and those who voluntarily agree to participate in the study were checked for eligibility. Participants' recruitment was carried out by distribution of public relations to local residents and 42 people participated in explanatory meeting at the community center. One of the authors (K. M.) provided for participants in the explanatory meeting a 30-minute lecture on nutrition and prevention of cognitive function decline. The lecture presented previous studies which had examined the effects of CGAs intake on endothelial function [16] and body fat [17]. A beverage with the same components had been used, although the amount consumed was different from the amount in this study. Also presented was the possibility of improvement in cognitive function, as demonstrated by animal experiment [12, 15]. After the lecture was over, we also explained the content of the experiment to be conducted in this study. In the explanation of the experiment content, the participants in the explanatory meeting were informed that it was uncertain whether all applicants would be able to become participants in the experiment, even if they were willing. After explaining the content of the experiment, written informed consent for participation was obtained, to apply for becoming subjects in the experiment. 28 participants agreed to participate in this study. Exclusion criteria were physical or mental disorder, visual impairment, and movement-related pain. In addition, for screening, participants completed Kihon Checklist, a self-administered questionnaire consisting of 25 questions in seven categories: instrumental activities of daily living, physical strength, nutritional status, oral function, cognitive function, and depression risk [18]. And those who checked at least one question in the cognitive function category (“Do others point out your forgetfulness or tell you ‘You always ask the same thing'?,” “When you want to make a call, do you usually search for the telephone number and call on your own?,” or “Do you sometimes not know what the date is?”) were considered eligible for enrollment. Finally, 8 participants (2 men, 6 women) were enrolled in this study and those who completed the 6-month trials were included in the analysis.

### 2.2. Materials

The test beverage for this study consisted of 330 mg CGAs dissolved in 100 mL of water and contained less than 10 kcal of energy. The CGAs were extracted from green coffee beans using a hot water extraction method and decaffeinated by activated carbon; then, a dry powder was obtained by spray drying [16]. The caffeine level of the test beverage was below the limit of quantification (<1 mg/100 g) to avoid its potential effects on cognitive functions and sleep qualities [10, 19]. The CGAs comprised 5-CQA, 3-CQA, 4-CQA, 3,4-diCQA, 3,5-diCQA, 4,5-diCQA, 3-FQA, 4-FQA, and 5-FQA and was assessed using high-performance liquid chromatography. In terms of composition, the CGAs consisted of 58.3% CQA (total of 3-CQA, 4-CQA, and 5-CQA), 19.9% feruloylquinic acid (total of 3-FQA, 4-FQA, and 5-FQA), and 21.8% dicaffeoylquinic acid (3,4-diCQA, 3,5-diCQA, and 4,5-diCQA).

### 2.3. Study Design

This study was approved by the Ethics Committee of Yonezawa University of Nutrition Sciences (Approval Number 26-3, May 28, 2014) and was conducted in accordance with the Declaration of Helsinki. In this single-arm study, participants ingested one 100 mL bottle of test beverage containing 330 mg of CGAs daily before bedtime during a 6-month period between August 2014 and May 2015. A 1-month supply of test beverages was delivered to the participants' homes every month by the research staff. A diary for recording the details of test beverage consumption and daily activities was collected for evaluation every 2 months, and neurocognitive tests were performed before and after the nutritional intervention period.

### 2.4. Measurement Items

#### 2.4.1. Participant Attributes

Participant attributes included physical characteristics (height, weight), education history, and Mini-Mental State Examination (MMSE) scores.

#### 2.4.2. Assessment of Cognitive Function

Cognitive function was assessed by using two types of computerized neurocognitive test batteries: Cogstate (Cogstate Ltd., Melbourne VIC, Australia) [20] and CNS Vital Signs (CNS Vital Signs LLC, Morrisville, NC) [21]. A practice test was run with each battery 1 week before the actual test.

*(1) Cogstate.* The Cogstate test battery consisted of 8 tests for different domains of cognitive function: the Groton Maze Chase Test for assessing visual motor function, Groton Maze Learning Test for executive function, Detection Test for psychomotor function, Identification Test for attention, One Back Test (OBT) and Two Back Test for working memory, One Card Learning Test for visual learning, and Continuous Paired Associate Learning Test for paired associate learning.

*(2) CNS Vital Signs.* The CNS Vital Signs test battery (basic package) consists of 7 tests for neurocognitive function: Verbal Memory Test (VBM), Visual Memory Test (VIM), Finger Tapping Test (FTT), Symbol Digit Coding (SDC), Stroop Test (ST), Shifting Attention Test (SAT), and Continuous Performance Test (CPT). As shown in Table 1, the scores on the 7 tests generate 11 domain scores: composite memory, verbal memory, visual memory, psychomotor speed, reaction time, complex attention, cognitive flexibility, processing speed, executive function, simple attention, and motor speed. For example, composite memory is calculated from the total number of correct hits and passes in the Verbal Memory Test and Visual Memory Test. The detailed calculation methods of the other domain scores are described in the previous study [21]. All scores are age-adjusted and standardized by setting the mean score to 100 and the standard deviation (SD) to 15. High scores indicate superior neurocognitive function.

#### 2.4.3. Blood Parameters

Blood was collected early in the morning during fasting. Measurements of cortisol, dehydroepiandrosterone sulfate (DHEA-S), brain-derived neurotrophic factor (BDNF), and A*β*40 and A*β*42 levels were performed by LSI Medience Corp. (Tokyo, Japan) and Cosmo Bio Co., Ltd. (Tokyo, Japan) according to conventional methods.

### 2.5. Statistical Analysis

Measurement data are expressed as mean and standard deviation (SD). Data obtained before and after the nutritional intervention were compared using the paired *t*-test, and effect size was calculated using Cohen's *d*  (*d*). In addition, parameters showing a significant difference after intervention were further analyzed to quantify the change and subjected to Pearson correlation analysis. Statistical analysis was performed using SPSS for Windows (version 22.0J; Tokyo, Japan), and significance was set at *p* < 0.05.

## 3. Results

### 3.1. Participants

Eight community-dwelling elderly individuals aged 71.5 ± 4.2 years fulfilled the inclusion criteria of this study. They had 12.6 ± 2.3 years of education and scored 26 points or higher on the MMSE (mean ± SD, 28.4 ± 1.4). The rate of ingestion of the test beverage was 88.6 ± 7.1%. Analysis of participant attributes revealed that mean preintervention and postintervention heights were 154.3 ± 7.8 cm and 153.7 ± 7.7 cm and preintervention and postintervention were 53.0 ± 8.3 kg and 53.4 ± 9.0 kg, respectively, with no significant change in height (*t*(7) = 2.291, *p* = 0.56, and *d* = 0.039) or weight (*t*(7) = −0.667, *p* = 0.526, and *d* = 0.050). Body mass index was 22.4 ± 3.4 kg/m^2^ preintervention and 22.6 ± 3.6 kg/m^2^ postintervention, with no significant difference (*t*(7) = −0.941, *p* = 0.378, and *d* = 0.075).

### 3.2. Cognitive Function

#### 3.2.1. Cogstate

The computerized neurocognitive Cogstate test battery consists of 8 tests of cognitive function.  Table 2 shows scores from the tests conducted before and after a 6-month nutritional intervention. In OBT, the total number of errors decreased significantly from 4.0 ± 4.3 preintervention to 1.1 ± 1.9 postintervention, while the accuracy increased significantly from 89.6 ± 10.2% to 96.7 ± 5.2%, respectively.

#### 3.2.2. CNS Vital Signs

*(1) Neurocognitive Tests.* Table 3 shows the results of another computerized neurocognitive test battery, CNS Vital Signs, conducted before and after the 6-month intervention period. The number of right taps during the FTT increased significantly from 47.6 ± 2.3 preintervention to 50.3 ± 3.7 postintervention, but the number of left taps tended to increase from 44.8 ± 5.2 to 46.9 ± 4.2, respectively. There were 25.4 ± 11.4 and 35.1 ± 13.1 correct responses to the SAT before and after intervention, respectively, with a significant increase after intervention. Conversely, the number of errors decreased significantly from 17.1 ± 8.4 preintervention to 9.6 ± 8.1 postintervention.

*(2) Neurocognitive Domains.* Table 4 shows the scores for 11 neurocognitive domains from the 7 CNS Vital Signs tests. The score for composite memory increased significantly from 77.4 ± 11.0 preintervention to 93.0 ± 18.8 postintervention. Similarly, the score for verbal memory increased significantly from 84.0 ± 10.6 preintervention to 103.4 ± 19.4 postintervention. In terms of complex attention, the score increased significantly from 88.5 ± 13.7 preintervention to 101.6 ± 13.7 postintervention. The score for cognitive flexibility was 82.3 ± 13.3 preintervention and 96.1 ± 14.2 postintervention, showing a significant increase after the 6-month intervention. Additionally, a significant postintervention increase was observed in executive function (82.8 ± 13.0 to 96.3 ± 13.7) and motor speed (92.5 ± 5.2 and 98.4 ± 7.7).

### 3.3. Blood Parameters

Table 5 shows the changes in blood parameters. After the intervention, there was a significant increase in serum DHEA-S levels and a significant decrease in plasma A*β* (1–42) and A*β*42/A*β*40 levels.

### 3.4. Correlation between Cognitive Function and Blood Parameters

**Figure 1:**
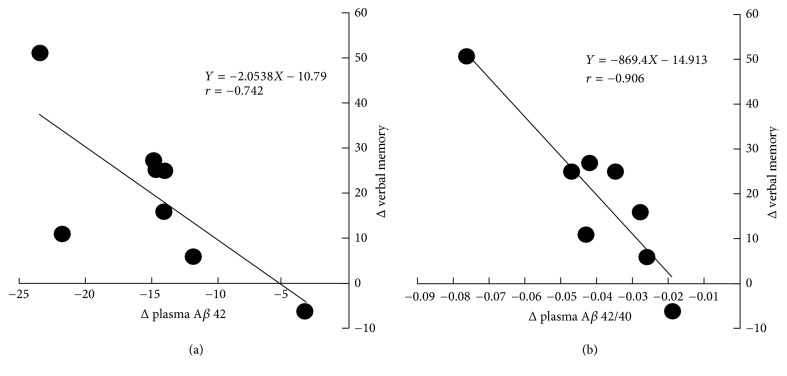
Correlation between Δverbal memory and Δplasma A*β* 42 (a) and plasma A*β* 42/40 (b).

A correlation analysis conducted between biological data and neurocognitive test items showed a significant change due to the intervention: a significant negative correlation between verbal memory and plasma A*β* (1–42) (*r* = −0.742, *p* = 0.035) and plasma A*β*42/A*β*40 (*r* = −0.906, *p* = 0.002) levels (Figure 1).

## 4. Discussion

To our knowledge, this is the first study to show that a 6-month intake of CGAs improves cognitive function in the elderly. The CGA-induced improvement in cognitive function was reflected in the computerized neurocognitive tests OBT, SAT, and FTT. Also, a significant improvement in composite and verbal memory, complex attention, cognitive flexibility, executive function, and motor speed was identified by combining scores from the individual CNS Vital Signs tests. Among the biological parameters, plasma A*β*42 and A*β*42/A*β*40 levels decreased significantly, whereas DHEA-S levels increased significantly. In particular, plasma A*β*42 and A*β*42/A*β*40 decreased to levels where improvement of verbal memory was apparent.

Using 2 computerized test batteries, 15 different neurocognitive tests were conducted in this study. The results showed an improvement in the OBT error response and accuracy, in the SAT correct and error responses, and in the FTT tap average. Previous studies have shown an association between OBT response and working memory [22] and between SAT/FTT performance and attentional and executive functions. Furthermore, cognitive function is associated with working memory and attentional and executive functions in the prefrontal region [23]. Therefore, CGAs are presumed to improve brain function in the prefrontal region.

Previous studies have demonstrated the antioxidant effects of CGAs [24] and the improvement in cognitive function mediated by CGAs through their antioxidative and neuroprotective effects. The antioxidant effects are considered one of the most important factors to prevent the age-related neurodegenerative diseases as recent studies show that the oxidative stress links to A*β* aggregation and following dementia due to AD [25]. In a study using mice with scopolamine-induced amnesia, administration of 5-CQA, the main component of CGAs, inhibited acetylcholinesterase activity in the prefrontal cortex [12]. Whether the results of the animal study can be applied to humans is unknown; nevertheless, the findings of the present study suggest that CGAs improve brain function in the prefrontal region in humans.

The metabolism of CGAs in the body generates gallic acid, caffeic acid, coumaric acid, ferulic acid, and sinapic acid [14], all of which have been shown to suppress the breakdown of the amyloid-precursor protein, which is pathologically related to the A*β* (1–42) protein, lipid peroxidation, and neurite extension of hippocampal neurons [13, 15]. These functions are likely involved in the improved memory following CGA intake.

We also analyzed the correlation between neurocognitive test scores and biological data and confirmed a significant decrease in plasma A*β*42 and A*β*42/A*β*40 levels and a significant increase in DHEA-S levels after a 6-month CGA intake period. Specifically, the change in plasma A*β*42 or A*β*42/A*β*40 levels was negatively correlated with an improvement in verbal memory (Figure 1), indicating that verbal memory improves as the level of A*β*42 or A*β*42/A*β*40 decreases. In previous cohort studies, the level of A*β*42 increased before the onset of AD or in the early stage of AD but decreased gradually thereafter [26–28]. Thus, the level of plasma A*β* is a predictor of AD and cognitive impairment. However, no study has investigated the effect of food or dietary intervention using plasma A*β* as an indicator. In a cross-sectional study of healthy elderly individuals aged ≥65 years with normal cognitive function, Gu et al. investigated the association between nutrient intake and plasma A*β* levels and reported that higher dietary intake of *ω*-3 PUFAs was associated with lower plasma levels of A*β*42 [29]. In another study of healthy elderly individuals aged ≥65 years, exercise intervention led to an improvement in cognitive function and a significant reduction in plasma A*β*42/A*β*40 levels. However, no correlation with improvement in cognitive function and no degree of change in A*β*42/A*β*40 levels was observed [30]. These findings suggest that although it is too soon to draw conclusions about plasma A*β*42/A*β*40 levels, the correlation between the improvement in verbal memory and the change in A*β*42/A*β*40 levels in this study warrants further investigation.

In this study, attentional, executive, and memory functions improved significantly after a 6-month CGA intake period in community-dwelling elderly individuals with complaints of subjective memory loss. Previous studies have shown that CGAs improve blood pressure and vascular endothelial functions, both of which are associated with the onset of dementia [17, 31, 32]. Because hypertension in middle age is a risk factor for dementia and cognitive impairment in old age [33], continuous consumption of CGAs may delay the onset of dementia.

This study has limitations as follows. First, this was a single-arm study with no control. Therefore, the beneficial effect observed in this study might have been due, at least in part, to placebo effects. The influences of the participants' anticipation and activities other than CGA intake cannot completely be ruled out although there seemed to be no great changes in their activities during the 6-month trial according to the diary they submitted. Second, multiple cognitive functions were carefully investigated in this study, but because of the small number of participants, further study with a larger number of participants and a control group is needed to verify the effect of CGA intake on cognitive function.

## 5. Conclusion

The findings of this study suggest that CGAs may improve cognitive function, especially attentional, executive, and memory functions in the elderly.

## Figures and Tables

**Table 1:** Cognitive domain scores in CNS Vital Signs.

Cognitive domains	Tests for score calculations
Composite memory	VBM + VIM
Verbal memory	VBM
Visual memory	VIM
Psychomotor speed	FTT + SDC
Reaction time	ST
Complex attention	ST + SAT + CPT
Cognitive flexibility	ST + SAT
Processing speed	SDC
Executive function	SAT
Simple attention	CPT
Motor speed	FTT

VBM: Verbal Memory Test, VIM: Visual Memory Test, FTT: Finger Tapping Test, SDC: Symbol Digit Cording, ST: Stroop Test, SAT: Shifting Attention Test, and CPT: Continuous Performance Test.

**Table 2:** Neurocognitive function (Cogstate).

Task	Pre	Post	*t*	*p* value	ES (*d*)
*Groton Maze Chase Test*					
Number of correct responses	23.0 ± 11.2	25.7 ± 8.6	0.797	0.456	0.271
Number of total errors	1.1 ± 1.5	2.7 ± 3.0	1.220	0.268	0.669
Correct responses per second	0.77 ± 0.37	0.86 ± 0.29	0.796	0.456	0.271
*Groton Maze Learning Test*					
Number of total errors	80.9 ± 25.0	89.7 ± 21.0	0.936	0.385	0.383
Correct responses per second	0.45 ± 0.12	0.41 ± 0.09	1.346	0.227	0.371
*Detection Test*					
Number of total errors	0.71 ± 1.10	0.86 ± 1.80	0.157	0.881	0.093
Reaction times for correct responses	433 ± 54	535 ± 142	2.248	0.066	0.951
Accuracy (%)	98.1 ± 2.9	97.8 ± 4.0	0.113	0.914	0.067
*Identification Test*					
Number of total errors	1.29 ± 1.25	1.86 ± 2.79	0.603	0.603	0.264
Reaction times for correct responses	698 ± 112	705 ± 79	0.219	0.834	0.072
Accuracy (%)	96.1 ± 3.8	94.7 ± 7.3	0.555	0.599	0.229
*One Card Learning Test*					
Number of total errors	34.0 ± 8.6	35.6 ± 11.3	1.010	0.352	0.157
Reaction times for correct responses	1094 ± 263	1073 ± 165	0.238	0.820	0.096
Accuracy (%)	61.6 ± 9.6	60.3 ± 12.1	0.763	0.474	0.118
*One Back Test*					
Number of total errors	4.0 ± 4.3	1.1 ± 1.9	2.547	0.044	0.865
Reaction times for correct responses	968 ± 171	925 ± 82	0.712	0.503	0.325
Accuracy (%)	89.6 ± 10.2	96.7 ± 5.2	2.765	0.033	0.871
*Two Back Test*					
Number of total errors	6.57 ± 4.43	5.57 ± 3.21	0.491	0.641	0.259
Reaction times for correct responses	1049 ± 251	1001 ± 161	0.618	0.559	0.227
Accuracy (%)	83.9 ± 9.4	85.7 ± 7.2	0.416	0.692	0.218
*Continuous Paired Associate Learning Test*					
Number of total errors	131 ± 60	125 ± 94	0.184	0.860	0.080
Reaction times for correct responses	5287.6 ± 1422.0	4620.7 ± 2222.7	0.874	0.416	0.431
Accuracy (%)	32.6 ± 10.1	39.1 ± 18.7	1.047	0.335	0.436

Pre: preintervention; post: postintervention; ES: effect size; the measurement unit of reaction time is millisecond.

**Table 3:** Neurocognitive function (CNS Vital Signs).

Task	Pre	Post	*t*	*p* value	ES (*d*)
*Verbal Memory Test*					
Correct hit immediate	11.4 ± 3.2	13.6 ± 0.9	1.913	0.097	0.967
Correct passes immediate	12.1 ± 3.2	12.6 ± 2.60	0.661	0.529	0.172
Correct hits delay	8.5 ± 5.6	11.9 ± 1.6	1.478	0.183	0.820
Correct passes delay	11.5 ± 3.3	12.1 ± 2.7	0.615	0.558	0.206
*Visual Memory Test*					
Correct hit immediate	11.1 ± 2.5	11.9 ± 3.2	0.622	0.554	0.263
Correct passes immediate	7.6 ± 3.8	9.1 ± 2.5	1.587	0.156	0.466
Correct hits delay	9.8 ± 4.5	9.6 ± 2.2	0.072	0.945	0.035
Correct passes delay	6.9 ± 3.8	8.0 ± 2.3	0.709	0.501	0.365
*Finger Tapping Test*					
Right taps average	47.6 ± 2.3	50.3 ± 3.7	2.420	0.046	0.844
Left taps average	44.8 ± 5.2	46.9 ± 4.2	2.323	0.053	0.451
*Symbol Digit Coding*					
Correct hits	36.0 ± 7.6	35.3 ± 9.2	0.281	0.787	0.089
Errors	1.8 ± 1.7	3.8 ± 3.2	1.313	0.231	0.784
*Stroop Test*					
Simple reaction time	566 ± 326	464 ± 93	1.072	0.319	0.430
Complex reaction time Correct	739 ± 203	886 ± 128	1.623	0.149	0.865
Stroop reaction time correct	1131 ± 289	1005 ± 138	1.367	0.214	0.557
Stroop commission errors	1.5 ± 0.5	1.9 ± 1.7	0.753	0.476	0.293
*Shift Attention Test*					
Correct responses	25.4 ± 11.4	35.1 ± 13.1	2.654	0.033	0.794
Errors	17.1 ± 8.4	9.6 ± 8.1	2.772	0.028	0.906
Correct reaction time	1311 ± 152	1241 ± 167	1.197	0.270	0.437
*Continuous Performance Test*					
Correct responses	39.9 ± 0.4	39.6 ± 0.7	0.798	0.451	0.429
Omission errors	0.13 ± 0.35	0.38 ± 0.74	0.798	0.451	0.429
Commission errors	0.75 ± 0.89	0.25 ± 0.71	1.080	0.316	0.624
Choice reaction time correct	535 ± 78	541 ± 44	0.268	0.796	0.087

Pre-: preintervention; post: postintervention; ES: effect size.

**Table 4:** Neurocognitive function (CNS Vital Signs).

Domain	Pre	Post	*t*	*p* value	ES (*d*)
Composite memory	77.4 ± 11.0	93.0 ± 18.8	2.434	0.045	1.016
Verbal memory	84.0 ± 10.6	103.4 ± 19.4	3.221	0.015	1.239
Visual memory	78.1 ± 12.2	84.3 ± 19.0	0.819	0.440	0.384
Psychomotor speed	90.3 ± 8.2	95.4 ± 9.4	1.323	0.228	0.581
Reaction time	76.1 ± 13.7	80.1 ± 12.0	0.933	0.382	0.311
Complex attention	88.5 ± 13.7	101.6 ± 13.7	2.918	0.022	0.957
Cognitive flexibility	82.3 ± 13.3	96.1 ± 14.2	2.943	0.022	1.009
Processing speed	92.4 ± 12.5	91.4 ± 10.1	0.164	0.874	0.088
Executive function	82.8 ± 13.0	96.3 ± 13.7	2.766	0.028	1.011
Simple attention	99.3 ± 8.4	104.3 ± 9.2	1.228	0.259	0.570
Motor speed	92.5 ± 5.2	98.4 ± 7.7	2.552	0.038	0.896

Pre: preintervention; post: postintervention; *t*: test; ES: effect size.

**Table 5:** Blood parameters.

	Pre	Post	*t*	*p* value	ES (*d*)
BDNF (ng/mL)	22.96 ± 5.12	21.90 ± 5.38	0.774	0.464	0.201
DHEA-S (*µ*g/dL)	55.88 ± 29.53	68.50 ± 39.89	2.583	0.036	0.360
Cortisol (*µ*g/dL)	9.15 ± 0.37	10.29 ± 3.79	0.846	0.426	0.344
Amyloid-*β* 40	360.00 ± 42.35	352.63 ± 43.50	0.768	0.468	0.172
Amyloid-*β* 42	52.90 ± 6.80	38.21 ± 4.49	6.762	<0.001	2.549
Amyloid-*β* 42/40	0.149 ± 0.025	0.109 ± 0.012	6.294	<0.001	1.973

Pre: preintervention; post: postintervention; ES: effect size.
